# Translation and cultural adaptation of the pectus excavatum evaluation questionnaire to Spanish and Catalan

**DOI:** 10.1186/s41687-022-00527-x

**Published:** 2022-12-02

**Authors:** Irene de Haro Jorge, Xavier Tarrado, Asteria Albert Cazalla, Natalie García-Smith, Alba Fernàndez-Candial, Carlos Salcedo-Gil

**Affiliations:** 1 Department of Pediatric Surgery, Hospital Sant Joan de Déu, Passeig Sant Joan de Déu 2, Esplugues de Llobregat, 08950 Barcelona, Spain; 2 Head of the Department of Pediatric Surgery, Hospital Sant Joan de Déu, Barcelona, Spain; 3 Department of Pediatrics, Hospital General Castelló, Castelló de La Plana, Spain; 4 Department of Communication, Blue Globe Media, Barcelona, Spain; 5 Department of Oral and Maxillofacial Surgery, Hospital Universitari de Son Espases, Palma, Spain

## Abstract

**Purpose:**

Pectus excavatum (PE) is the most common congenital chest wall deformity. It can have a negative effect in exercise tolerance. However, cosmetic features are the most frequent concerns in these patients. The PE evaluation questionnaire (PEEQ) is a patient reported outcome (PRO) tool to measure the physical and psychosocial quality-of-life changes after surgical repair of PE. No specific tool has been developed in our languages to evaluate PRO in PE patients. Our aim is to translate and culturally adapt the PEEQ to Spanish and Catalan.

**Methods:**

Guidelines for translation of PRO were followed. The PEEQ, consisting of 34 items, was translated from English to Spanish and to Catalan. Three forward translations and one back translation were performed for each language. Cognitive debriefing interviews were developed.

**Results:**

The reconciliation of the forward translations revealed a 14.7% of inconsistencies for each language. The Spanish back translation showed a 64.7% of disagreement with the source, the Catalan 58.8%. Changes in each reconciled version were made to amend the diverting items. Cognitive debriefing: Catalan version: 15 participants, 10 males, 5 patients had been operated. 12 patients showed difficulties for understanding 4 of the items. Spanish version: 17 participants, 11 males, 5 had been operated. 13 patients showed difficulties for understanding 4 of the items. We made modifications of the problematic items, in order to make them easier to understand for our patients. We tested the last version in a new group of patients. Catalan: 7 patients, 5 males. One patient showed difficulties for understanding item 11, so we added a further clarification of this item. Spanish: 7 patients, all males. There were any difficulties for understanding.

**Conclusion:**

After a thorough process of translation and cultural adaptation, we reached a Catalan and a Spanish version of PEEQ. This work constitutes the first step to reach a specific PE PRO tool in our languages. However, it needs to be validated, with a higher number of patients, before being widely used in a clinical setting.

## Background

*Pectus excavatum* (PE) is the most common congenital chest wall deformity. It is defined by a depression of the sternal body and the lower costal cartilages. Its prevalence is 1–8/1.000 children [1] being more frequent in boys [2]. The sternal depression may restrict thoracic volume and cause cardiac compression; having a negative effect in exercise tolerance [3]. Cosmetic features are also a frequent concern [4, 5], and several studies have shown the repercussion of the defect in the psychological wellbeing of children with PE [6].

The PE evaluation questionnaire (PEEQ) was developed and validated by Lawson et al. [7] as a patient reported outcome (PRO) tool to measure the physical and psychosocial changes after surgical repair of PE. So far, no specific tool has been developed in Catalan or Spanish, to evaluate PRO in children with PE. The aim of our study is to translate and culturally adapt the PEEQ to Catalan and Spanish.

## Material and methods

The study was evaluated and approved by the Research Ethics Board of Hospital Sant Joan de Déu (PIC-82–19) complying with local laws on Biomedical Research. Legal guardians and patients older than 18 years provided written informed consent. The PEEQ consists of both preoperative and postoperative telephone questionnaires [7]. It includes questions related to body image and physical activities. Answers are given using a Likert-type scale, reflecting the extent or frequency of a particular experience. Higher values on the response scale indicate a less desirable experience. The questionnaire has one part for the patients and another one for the parents. The patient’s preoperative part of the questionnaire consists of 15 items; the postoperative part consists of 17 items. The parents’ part consists of 16 items; there is only a slight difference in one item that was considered irrelevant for the analysis. The items are included in Tables 1, 2.

**Table 1:** English to Spanish translation process

Type	Item	3 discrepancies in forward translation	Forward translation explanation	Changed meaning post back translation	Solution	Difficulties for patient to understand	Solution
1. Child	How do you feel about the way you look in general?	No	–	Yes (about your appearance in general?)	Keep the Spanish equivalent for “about your appearance in general?”	No	–
2. Child	How do you feel about the way you look with your shirt or your top off?	No	–	Yes (when you don't wear a shirt?)	Change for Spanish equivalent for “when you are with your shirt or your top off”	No	–
3. Child	If you had to spend the rest of your life with your chest as it looks now, how would you feel?	No	–	No (with your chest as you have it now?)	–	No	–
4. Child	How often do other kids do fun about you because of your chest?	No	Change for Spanish equivalent for “laugh at you”	Yes (the shape of your chest?)	Change again for Spanish equivalent for “do fun about you” and omit “the shape”	No	–
5. Child	How often do you avoid doing things, like spending the night at a friend’s house, because of the way your chest looks?	No	Change “doing things” for Spanish equivalent for “making plans”	Yes (Do you avoid making plans with your friends, like sleep over at their house; because of the shape of your chest?)	Change again for Spanish equivalent for “doing things”	1	–
6. Child	How often do you try to hide your chest to keep people from knowing about it?	No	–	Yes (so that people do not know how you have it?)	Keep the Spanish equivalent for “people do not know how you have it?”	No	–
7. Child	How often are you bothered because of the way your chest looks?	No	Use the Spanish equivalence for “worried” instead of “bothered”	Yes (Are you worried about the appearance of your chest?)	Change again for the Spanish equivalent for “are you bothered”	2	Add the Spanish equivalent for “by other people”
8. Child	How often does your chest make you feel shy or self-conscious?	Yes (shy)	Exclude “shy” from Spanish version	Yes (Do you feel ashamed about the shape of your chest?)	Keep the Spanish equivalent for “self-conscious”	7	Add again the Spanish equivalent for “shy”
9. Child	How often do you feel bad about yourself because of the way your chest looks?	No	–	Yes (Does the appearance of your chest make you feel bad?)	–	No	–
10. Child	Have trouble running around or exercising because it made your chest hurt?	No	–	No (Problems when running or exercising because your chest hurt)	–	No	–
11. Child	Have shortness of breath	Yes	Use the Spanish equivalent for “feeling shortness of breath”	No (Feeling short of breath)	Remove the Spanish equivalent for “feeling”	3	Add the Spanish equivalent for “breathing problems”
12. Child	Be tired	No	–	No	–	No	–
13. Child	Not be able to participate in gym class	No	Change for the Spanish equivalent for “to do physical education class”	Yes (to do physical education class)	Change for the Spanish equivalent for “participate”	No	–
14. Child	Miss school	No	–	No	–	No	–
15. Child before surgery	How much do you want the surgery to make your chest look different?	Yes	Use the Spanish equivalent for “would you like to undergo surgery to change how your chest looks?”	Yes (Would you like to operate to have a different breast?)	Keep the Spanish equivalent for “would you like to undergo surgery to change how your chest looks?”	No	–
15. Child after surgery	How did the surgery change how your chest looks?	No	–	Yes (Do you think the operation has changed the appearance of your chest?)	Keep the Spanish equivalent for “Do you think the operation has changed the appearance of your chest?”	No	–
16. Child after surgery	How did the surgery change how you feel about your chest?	No	–	Yes (After the operation, how do you feel about your chest?)	Keep the Spanish equivalent for “After the operation, how do you feel about your chest?”	No	–
17. Child after surgery	How happy are you that you had the surgery?	No	–	No (Are you happy to have gone through the surgery?)		No	–
1. Parents	How often has your child’s pectus excavatum caused him/her to have trouble being physically active?	No	Change for the Spanish equivalent for “difficulties in physical activity”	Yes (Difficulties in physical activity)	Change for the Spanish equivalent for “trouble”	No	–
2. Parents	How often has your child’s pectus excavatum caused him/her to have chest pain when physically active, such as running or playing sports?	No	–	No (Chest pain during physical activity -running, playing sports-)	–	No	–
3. Parents	How often has your child’s pectus excavatum caused him/her to have shortness of breath?	No	Add the Spanish equivalent for “feeling”	Yes (Feeling short of breath)	Omit the equivalent for “feeling” in the Spanish version	No	–
4. Parents	How often has your child’s pectus excavatum caused him/her to become tired?	No	–	No (tiredness)		No	–
5. Parents	How often has your child’s pectus excavatum caused him/her to have problems gaining weight?	No	–	No (Problems to gain weight)	–	No	–
6. Parents	How often has your child’s pectus excavatum caused him/her to be irritable?	No	Change for the Spanish equivalent for “irritability”	No (Irritability)		No	–
7. Parents	How often has your child’s pectus excavatum caused him/her to be frustrated?	No	Add the Spanish equivalent for “feeling”	Yes (Feeling frustrated)	Omit the equivalent for “feeling” in the Spanish version	No	–
8. Parents	How often has your child’s pectus excavatum caused him/her to be sad or depressed?	No	Add the Spanish equivalent for “feeling”	Yes (Feeling sad or depressed)	Omit the equivalent for “feeling” in the Spanish version	No	–
9. Parents	How often has your child’s pectus excavatum caused him/her to be restless?	No	Add the Spanish equivalent for “sensation”	Yes (Sensation of anxiety)	Omit the equivalent for “sensation” and keep the Spanish equivalent for “restlessness” in the Spanish version	No	–
10. Parents	How often has your child’s pectus excavatum caused him/her to be isolated?	No	Add the Spanish equivalent for “sensation”	Yes (Sensation of isolation)	Omit the equivalent for “sensation” in the Spanish version	No	–
11. Parents	How often has your child’s pectus excavatum caused him/her to be made fun of?	No	Change for the Spanish equivalent for “be the object of jokes”	Yes (Be the object of jokes)	Change for the Spanish equivalent for “be made fun of” or “be mocked”	No	–
12. Parents	How often has your child’s pectus excavatum caused him/her to limit him/her playing sports?	No	–	Yes (Limitation to practise sport)	Keep the Spanish equivalent for “limitation to practise sport”	No	–
13. Parents	How often has your child’s pectus excavatum caused him/her to miss school	No	Change for the Spanish equivalent for “class”	Yes (Missing class)	Change “class” for the Spanish equivalent for “school”	No	–
14. Parents	How often has your child’s pectus excavatum caused him/her to be reluctant to wear a bathing suit?	Yes (reluctant)	Keep the Spanish equivalent for reluctant	No (Show reluctance to use a swimsuit)	–	No	–
15. Parents	How often has your child’s pectus excavatum caused him/her to be reluctant to change clothes in front of others?	Yes (reluctant)	Keep the Spanish equivalent for reluctant	No (Show reluctance to change clothes in front of others)	–	No	–
16. Parents	How often have you been concerned about the effects pectus excavatum has on your child’s life?	No	–	No (Are you concerned about the effects of pectus excavatum on your child's life?)	–	No	–

**Table 2:** English to Catalan translation process

Type	Item	3 discrepancies in forward translation	Translation explanation	Changed meaning post back translation	Solution	Difficulties for patient to understand	Solution
1. Child	How do you feel about the way you look in general?	No	–	Yes (your general appearance?)	–	2	Add the Catalan equivalent for “physical appearance”
2. Child	How do you feel about the way you look with your shirt or your top off?	No	–	Yes (when you aren’t wearing a T-shirt?)	Change for Catalan equivalent for “when you are with your shirt or your top off”	No	-
3. Child	If you had to spend the rest of your life with your chest as it looks now, how would you feel?	No	–	No (How would you feel if you had to live the rest of your life with your chest as it is now?)	-	No	–
4. Child	How often do other kids do fun about you because of your chest?	No	Change for Catalan equivalent for “laugh at you” and add the “appearance of your chest”	Yes (laugh at you)	Change again for Catalan equivalent for “be made fun of” or “be mocked” and omit the “appearance of your chest”	No	–
5. Child	How often do you avoid doing things, like spending the night at a friend’s house, because of the way your chest looks?	No	Change “doing things” for Catalan equivalent for “making plans”	Yes (Do you stop making plans with your friends, like a sleepover, because of your chest?)	Change again for Catalan equivalent for “doing things”	No	–
6. Child	How often do you try to hide your chest to keep people from knowing about it?	No	–	Yes (Do you try to hide your chest so that people don’t know what it looks like?)	-	No	–
7. Child	How often are you bothered because of the way your chest looks?	No	Use the Catalan equivalence for worried instead of bothered	Yes (Do you worry about how your chest looks?)	Change again for the Catalan equivalent for “are you bothered”	2	Add the Catalan equivalent for “by other people”
8. Child	How often does your chest make you feel shy or self-conscious?	No	Exclude “shy” from Catalan version	Yes (Do you have a complex about your chest?)	Keep the Catalan equivalent for “self-conscious”	7	Add again the Catalan equivalent for “shy”
9. Child	How often do you feel bad about yourself because of the way your chest looks?	No	–	No (Does the look of your chest make you feel bad?)	–	No	–
10. Child	Have trouble running around or exercising because it made your chest hurt?	No	–	No (Problems during running or exercising because your chest hurt)	–	No	–
11. Child	Have shortness of breath	No	Use the Catalan equivalent for “feeling shortness of breath”	No (Shortness of breath)	Omit the Catalan equivalent for “feeling”	1	Add the Catalan equivalent for “breathing problems”
12. Child	Be tired	No	–	No (Tiredness)	–	No	–
13. Child	Not be able to participate in gym class	No	Change “participate” for the Catalan equivalent for “do”	Yes (to follow the Physical Education class at school)	Keep the Catalan equivalent for “do”	No	–
14. Child	Miss school	No	–	No (Missing school)	–	No	–
15. Child before surgery	How much do you want the surgery to make your chest look different?	No	Use the Catalan equivalent for “would you like to undergo surgery to change how your chest looks?”	Yes (Would you like to be operated on so that your chest looks different?)	Keep the Catalan equivalent for “would you like to undergo surgery to change how your chest looks”	No	–
15. Child after surgery	How did the surgery change how your chest looks?	No	Use the Catalan equivalent for “do you think that the surgery changed the appearance of your chest?”	Yes (Do you think surgery has changed the appearance of your chest?)	Change for the Catalan equivalent for “how has the surgery changed the appearance of your chest?”	No	–
16. Child after surgery	How did the surgery change how you feel about your chest?	No	Use the Catalan equivalent for “After the surgery, how do you feel about your chest?”	Yes (After surgery, how do you feel about your chest?)	Keep the Catalan equivalent for “After the surgery, how do you feel about your chest?”	No	–
17. Child after surgery	How happy are you that you had the surgery?	No	Use the Catalan equivalent for “Are you happy that you had the surgery?”	Yes (Are you happy that you were operated on?)	Keep the Catalan equivalent for “Are you happy that you had the surgery?”	No	–
1. Parents	How often has your child’s pectus excavatum caused him/her to have trouble being physically active?	Yes	Change for Catalan equivalent for “difficulties to do physical activity”	Yes (Difficulties for exercising)	Change for the Catalan equivalent for “trouble”	No	–
2. Parents	How often has your child’s pectus excavatum caused him/her to have chest pain when physically active, such as running or playing sports?	No	–	Yes (Chest pain during exercise -running-)	Keep the Catalan equivalent for “chest pain during physical activity”		–
3. Parents	How often has your child’s pectus excavatum caused him/her to have shortness of breath?	Yes	Use Catalan equivalent for “feeling shortness of breath”	No (Shortness of breath)	Omit the equivalent for “feeling” in the Catalan version	No	–
4. Parents	How often has your child’s pectus excavatum caused him/her to become tired?	Yes	Use the Catalan equivalent for “tiredness”	No (tiredness)	Keep the Catalan equivalent for “tiredness”	No	–
5. Parents	How often has your child’s pectus excavatum caused him/her to have problems gaining weight?	No		Yes (Difficulties to gain weight)	Keep the Catalan equivalent for “problems to gain weight”	No	–
6. Parents	How often has your child’s pectus excavatum caused him/her to be irritable?	No	Use the Catalan equivalent for “feeling of irritability”	No (Irritability)	Omit the equivalent for “feeling” in the Catalan version	No	–
7. Parents	How often has your child’s pectus excavatum caused him/her to be frustrated?	No	Use the Catalan equivalent for “feeling of frustration”	No (Frustration)	Omit the equivalent for “feeling” in the Catalan version	No	–
8. Parents	How often has your child’s pectus excavatum caused him/her to be sad or depressed?	No	Use the Catalan equivalent for “feeling sadness or depression”	No (Sadness or depression)	Omit the equivalent for “feeling” in the Catalan version	No	–
9. Parents	How often has your child’s pectus excavatum caused him/her to be restless?	No	–	Yes (Worry)	Keep the Catalan equivalent for “restlessness”	No	–
10. Parents	How often has your child’s pectus excavatum caused him/her to be isolated?	No	Use the Catalan equivalent for “feeling of isolation”	Yes (Feeling of isolation)	Omit the equivalent for “feeling” in the Catalan version	No	–
11. Parents	How often has your child’s pectus excavatum caused him/her to be made fun of?	No	Use the Catalan equivalent for “other children laugh at him/her”	Yes (Other children to laugh at him/her)	Change for the Catalan equivalent for “be made fun of” or “be mocked”	No	–
12. Parents	How often has your child’s pectus excavatum caused him/her to limit him/her playing sports?	No	–	Yes (Limitation to practice sport)	Keep the Catalan equivalent for “limitation to practice sports”	No	–
13. Parents	How often has your child’s pectus excavatum caused him/her to miss school	No	Use the Catalan equivalent for “missing class”	Yes (Missing class)	Change “class” for the Catalan equivalent for “school”	No	–
14. Parents	How often has your child’s pectus excavatum caused him/her to be reluctant to wear a bathing suit?	Yes (reluctant)	Keep the Catalan equivalent for reluctant	No (To be reluctant to use a swimsuit)	–	No	–
15. Parents	How often has your child’s pectus excavatum caused him/her to be reluctant to change clothes in front of others?	Yes (reluctant)	Keep the Catalan equivalent for reluctant	No (To be reluctant to change clothes in front of other people)	–	No	–
16. Parents pre surg	How often have you been concerned about the effects pectus excavatum has on your child’s life?	No	–	No (Are you concerned about the effects of pectus excavatum on your child's life?)	–	No	–

### Translation and cultural adaptation of the PEEQ into Catalan and Spanish

Principles of good practice for the translation and cultural adaptation process for PRO were followed according to the International Society for Pharmacoeconomics and Outcomes Research (ISPOR) recommendations [8].

1. Preparation
  Developers provided the original version of PEEQ and gave permission to transform the telephonic version into a written questionnaire.
2. Forward translation
  Forward translators being native speakers of both target languages and having an advanced knowledge of English, were selected. One being a professional translator; and the other two individuals being pediatric surgeons—one with special dedication to PE patients and the other with previous experience in PRO translation [9]. A document including our written adaptation of the PEEQ, an explanation of the tool and the goals of our study was delivered to translators. It was stressed that the translation should be conceptual rather than literal. Common language was recommended, in order that the participants could easily understand the survey. Each translator developed a Catalan and a Spanish version of the PEEQ.
3. Reconciliation
  Reconciliation of the three forward translations into a single one, in each language, was performed in a meeting between the forward translators and the thoracic surgeon of our department. Agreement on key words and sentence construction was recorded for each item. An item was considered discordant if all 3 versions were different in one of the parts of the translation (key-word or sentence construction).
4. Back translation
  A native English speaker was the back translator for each target language, being both medical doctors. A document including the reconciled version, an explanation of the tool and the goals of the study was delivered to each translator. Again, it was stressed that the translation should be conceptual rather than literal and that the common language was recommended.
5. Back translation review
  A review of back translations was carried out by two forward translators and the thoracic surgeon. For each item, we recorded differences in the word use or sentence construction. Discrepancies between the original version and back translations were identified and an improved version was developed for each language. Despite detecting differences with the original source, the forward version was only changed in case there was a significant meaning alteration.
6. Harmonization
  Comparison between Catalan and Spanish versions and the original version was performed. Questions formulation was unified, at this point. This issue was amended in the back-translation review meeting.
7. Cognitive debriefing
  Patients with PE aged 8–21 years were selected from the thoracic outpatient agenda between 2019 and 2021. Patients who had undergone Nuss procedure and a group of untreated patients, were selected. The main researcher approached potential participants, explained the purpose of the study and invited them to participate. In an interview meeting with the main researcher, patients and parents were asked to answer the questionnaires and say if they had had any problem at understanding each item. Items requiring clarification were considered difficult to understand. Age, gender and PE treatment of the participants were recorded.
8. Review of the results of cognitive debriefing and finalization
  Sentences that were not easily understood were checked in a meeting between forward translators and the thoracic pediatric surgeon. Appropriate modifications were done to improve the final versions. In order to confirm that the modifications applied, achieved the understanding of the questionnaire, a subsequent cognitive debriefing was performed.
9. Proofreading
  Two other pediatric surgeons proofread the final versions.
10. Final report
  A final report was done including a description of all decisions regarding translation and cultural adaptation.

**Fig. 1:**
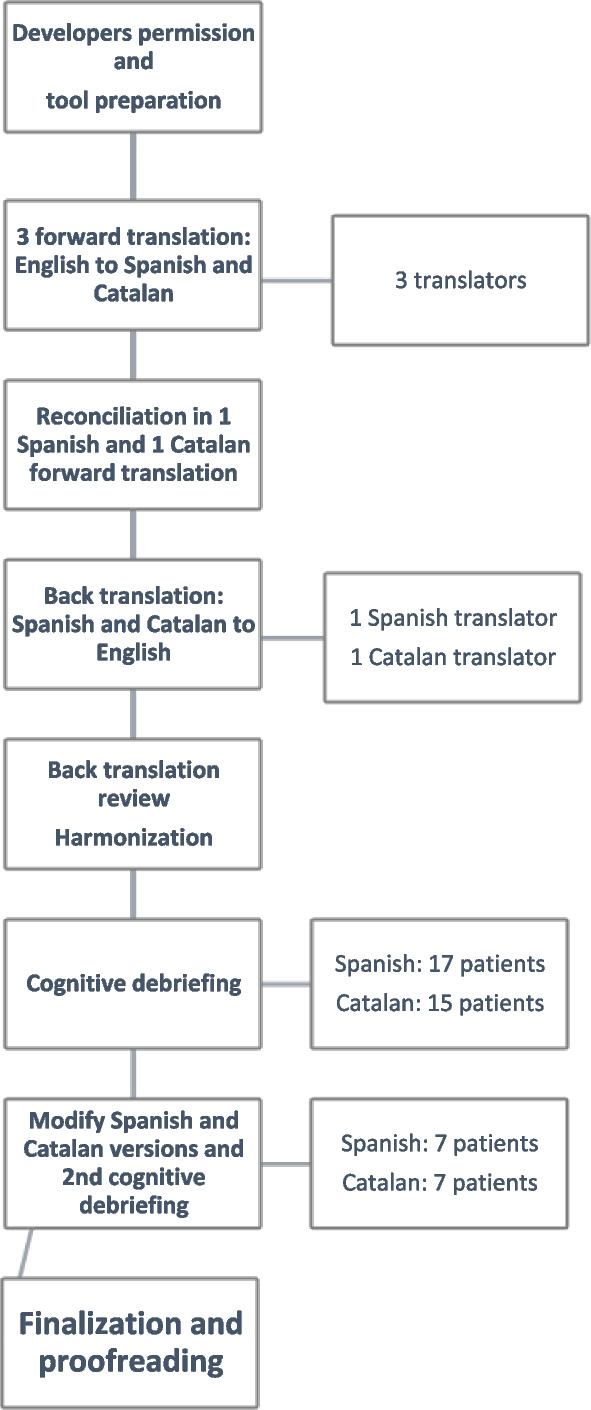
Flow diagram

A flow diagram of the process is depicted in Fig. 1.

## Results

Translation and cultural adaptation of the PEEQ resulted in the development of a Catalan and a Spanish version of the questionnaire.

### Forward translation

An evaluation of the degree of discordance between the three forward versions showed:

- Catalan: 14.7% of the items were discordant.
- Spanish: 14.7% of the items were discordant.

For each item, the best translation or a new one was chosen using words from any of the forward versions. In the case that two translations were equal and one was different, the equal ones were often selected for the reconciled version.

### Back translation

After reviewing the back translation, some items of the forward translation were changed in order to make the target language version more similar to the original source. In other cases, despite detecting differences, the forward translation was considered more accurate to the original source than the back translation; therefore, it wasn't changed.

- Catalan: wording or sentence construction changed in 58.8% of the items. However, only 41.2% of the cases were changed in the final version.
- Spanish: wording or sentence construction changed in 64.7% of the items. However, only 41.2% of the cases were changed in the final version.

### Harmonization

No major differences were found when comparing Catalan and Spanish versions. In both languages, the word “*forma*” (shape) was changed to “*aspecte*” or “*aspecto*” (appearance) in items 8 in order to match items 5, 7 and 9.

### Participants and results of cognitive debriefing

#### Catalan

Mean age of 15 participants was 14.5 years old, 10 males. Five participants had been operated. Parents did not show any difficulties at understanding, whereas patients showed lack of understanding: item 1 (2 patients), item 7 (2), item 8 (7) and item 11 (1). We added a clarification in item 1, 7 and 8. We tested the new version with 7 patients, 5 males. The mean age was 16 years old. Only one showed problems in understanding item 11. A clarification in item 11 was also added.

#### Spanish

Mean age of 17 participants was 15.4 years old, 11 males. Five participants had been operated. Parents did not show any difficulties at understanding, whereas patients showed lack of comprehension: item 5 (1 patient), item 7 (2), item 8 (7) and item 11 (3). We added a clarification in item 7, 8 and 11. We tested the new version in 7 patients, all males. The mean age was 12.9 years old. None of the patients or parents showed difficulties in understanding.

### Adaptation and translation process

Tables 1, 2 show the discordances found between forward and back translations, as well as the changes and solutions applied in order to get a translation that was equivalent to the source.

## Discussion

Patients with PE are frequently considered to have only a cosmetic problem, being often denied the opportunity for surgical correction. However, previous work on this subject has brought to light that surgical repair of PE improves body image and physical activity in these patients. Furthermore, scientific evidence has failed to prove a correlation between the anatomic severity of the chest depression and the PEEQ score, suggesting that the sole presence of the deformity, produces body image and psychosocial concerns. Therefore, it is more than obvious that the exclusive use of anatomic severity criteria to discern which patients should undergo surgical repair, is insufficient [10]. This fact stresses the need of having a validated tool in the patients’ own languages, that allows medical doctors to adequately evaluate the body image and physical difficulties that concern patients with PE.

In order to obtain an equivalent to the source and an adequate translation of PEEQ, all the steps of the ISPOR guidelines were followed. This detailed process can be used as an example to translate other PRO instruments.

We found around 15% discordances comparing forward translations. These discrepancies were amended in the reconciliation process, leading to a single reconciled version. The back-translation review revealed that in both languages, the sentence construction or wording used by back translators were different to the original source in more than half of the items. However, in spite of using differing words, the meaning of the translation was not always altered. To comply with the principle of giving prevalence to conceptual translation over literal translation, not every difference detected resulted in a modification of the reconciled version.

Most publications addressing translation and cultural adaptation of PRO instruments show a high variability in the number of discordances in forward and back translations [9, 11–15]. Furthermore, the lack of consensus analyzing the differences found on the translated versions, makes difficult to compare the results of different studies. Despite discordances in the translation process, the exhaustive translation method proposed by the ISPOR guarantees that the final translation is culturally equivalent to the original source [8].

There is no agreement in which method should be the preferred for harmonization. As previously described above, this issue was amended in the back-translation review meeting. There was high equivalence between both translations, probably due to the fact that the same translators developed forward translations in both languages. At that moment, the use of different words was detected to express the same concept in two different items. This issue was solved during harmonization process, where the same words were used in both translations, making both language versions more homogeneous.

A potential limitation of the present study is that the patients sample needed for cognitive interviews may be too small to be representative of our PE population. Therefore, responses to the questionnaire have been overlooked in this study. Also, the number of female participants is lower than males. However, the gender proportion in both samples (Catalan and Spanish) approaches that of PE population [2].

As far as we know, there is no other translation of the PEEQ following the ISPOR recommendations. Furthermore, until now there isn’t any other PRO tool in our languages for the evaluation of patients with PE. In the present study, an exhaustive process of translation and cultural adaptation was followed. This resulted in the development of a Catalan and a Spanish culturally appropriated version of the PEEQ. It represents the very first step to the process of producing an equivalent version of the instrument in both languages. Further studies including a higher number of patients, need to be done in order to validate the questionnaires.

## Conclusion

After a thorough process of translation and cultural adaptation, we produced a Catalan and a Spanish version of PEEQ. These versions need to be validated, with a higher number of patients, before being widely used in a clinical setting.

## Data Availability

The datasets used and/or analysed during the current study are available from the corresponding author on reasonable request.

## Abbreviations

PE: Pectus excavatum
PEEQ: Pectus excavatum evaluation questionnaire
PRO: Patient reported outcomes
ISPOR: International Society for Pharmacoeconomics and Outcomes Research

## References

[1] Kelly RE, Lawson ML, Paidas CN, Hruban RH (2005). Pectus excavatum in a 112-year autopsy series: Anatomic findings and the effect on survival. J Ped Surg..

[2] Oncel M, Sunam GS, Yildiran H (1988). Surgical repair of pectus excavatum. J Ped Surg.

[3] Kolvekar SK, Kolvekar S, Pilegaard H (2016). Introduction. Chest wall deformities and corrective procedures.

[4] Shamberger RC (2000). Cardiopulmonary effects of anterior chest wall deformities. Chest Surg Clin.

[5] Einsiedel E, Clausner A (2000). Funnel chest. Psychological and psychosomatic aspects in children, youngsters, and young adults. Minerva Pneumol..

[6] Colombani PM (2009). Preoperative assessment of chest wall deformities. Semin Thorac Cardiov.

[7] Lawson ML, Cash TF, Akers R, Vasser E, Burke B, Tabangin M (2003). A pilot study of the impact of surgical repair on disease-specific quality of life among patients with pectus excavatum. J Ped Surg.

[8] Wild D, Grove A, Martin M, Eremenco S, McElroy S, Verjee-Lorenz A (2005). Principles of good practice for the translation and cultural adaptation process for patient-reported outcomes (PRO) measures: report of the ISPOR task force for translation and cultural adaptation. Value Health.

[9] Tsangaris E, Riff KWYW, Vargas F, Aguilera MP, Alarcón MM, Cazalla AA (2017). Translation and cultural adaptation of the CLEFT-Q for use in Colombia, Chile, and Spain. Health Qual Life Outcomes.

[10] Kelly RE, Cash TF, Shamberger RC, Mitchell KK, Mellins RB, Lawson ML (2008). Surgical repair of pectus excavatum markedly improves body image and perceived ability for physical activity: multicenter study. Pediatrics.

[11] Sala-Sastre N, Herdman M, Navarro L, de la Prada M, Pujol R, Serra C (2010). Occupational dermatoses: cross-cultural adaptation of the Nordic occupational skin questionnaire (NOSQ-2002) From English to Spanish and Catalan. Actas dermo-sifiliogr.

[12] Rofail D, Acquadro C, Izquierdo C, Regnault A, Zarit SH (2015). Cross-cultural adaptation of the Schizophrenia Caregiver Questionnaire (SCQ) and the Caregiver Global Impression (CaGI) scales in 11 languages. Health Qual Life Outcomes.

[13] Poulsen L, Rose M, Klassen A, Roessler KK, Sørensen JA (2017). Danish translation and linguistic validation of the BODY-Q: a description of the process. Eur J Plast Surg.

[14] García-Díez I, Curto-Barredo L, Weller K, Pujol RM, Maurer M, Giménez-Arnau AM (2015). Adaptación transcultural del cuestionario Urticaria Control Test del alemán al castellano. Actas dermo-sifiliogr.

[15] Jones-Caballero M, Peñas PF, Garía-Díez A, Badía X, Chren MM (2000). The Spanish version of Skindex-29. Int J Dermatol.

